# Prevalence and characteristics of gastrointestinal disorders, medication use, and diagnostic interventions in pediatric patients with cystic fibrosis: a nested case–control analysis from the TriNetX EMR-derived global research network real-world dataset

**DOI:** 10.3389/fgstr.2024.1489876

**Published:** 2025-01-24

**Authors:** Carolena Trocchia, Maua Mosha, Bailey Hamner, Neil Goldenberg, Racha Khalaf

**Affiliations:** ^1^ School of Medicine, Stanford University, Stanford, CA, United States; ^2^ Department of Pediatric Gastroenterology, Hepatology and Nutrition, Stanford Healthcare, Stanford, CA, United States; ^3^ Institute for Clinical and Translational Research, Johns Hopkins All Children’s Hospital, Saint Petersburg, FL, United States; ^4^ Department of Pediatric Gastroenterology, Hepatology and Nutrition, University of South Florida, Tampa, FL, United States

**Keywords:** cystic fibrosis, exocrine pancreatic insufficiency, elexacaftor/tezacaftor/ivacaftor (ETI), modulator therapy, gastrointestinal disease

## Abstract

**Background:**

Gastrointestinal (GI) disease in pediatric patients with cystic fibrosis (CF) is a growing concern in the era of improved lung disease; however, the true prevalence of GI diagnoses, medical therapies, and frequency of diagnostic and therapeutic interventions in this population have yet to be explored on a multisystem scale. The aim of the present study was to describe, among pediatric patients with CF (PwCF) compared to a large cohort of healthy controls, the prevalence and types of 1) GI disorders; 2) GI medication use; and 3) GI procedural interventions.

**Methods:**

This was a multicenter case-control analysis using the TriNetX electronic medical record (EMR) global anonymized data platform. Patients were included if they had an ICD9/10 diagnosis code and were between the age of zero to ≤ 21 years between January 1st, 2010, and January 1st, 2020. Those with a history of solid organ transplants were excluded.

**Results:**

The cohort was comprised of 7,649 patients with a diagnosis of CF (cases) and 22,516,240 patients without CF (controls). The prevalence of any GI disorder was greater in pediatric PwCF compared to those without CF (73% versus <1%). The prevalence of diseases of the biliary tract and pancreas (43% vs. <1%; *p*<.0001), hepatic disease (8.8% vs. <1%; *p*<.0001), disease of the esophagus to duodenum (34% vs. <1%; *p*<.0001), and intestinal disease (43% vs. <1%; *p*<.0001) were each significantly greater in PwCF (*p*<0.0001). Furthermore, the frequencies of esophagogastroduodenoscopy (EGD), colonoscopy and endoscopic retrograde cholangiopancreatography (ERCP) were significantly higher among PwCF than controls (9.5% vs. <1%; 2.8%% vs. <1%; and 0.3% vs <.001% respectively; *p*<0.0001 for all comparisons).

**Conclusion:**

The prevalence of GI diagnoses, use of GI medications, and frequency of GI procedures are all higher among pediatric patients PwCF. Prospective multicenter studies are warranted to substantiate these findings, further investigate risk factors for GI disorders, and to describe potential changes in GI disorders with novel CF disease-modifying therapies.

## Introduction

Cystic fibrosis (CF) is a chronic disease with a pediatric onset most notably characterized by sinopulmonary and pancreatic complications but also involves gastrointestinal (GI) disorders (1–4). GI manifestations extend beyond the lumen and include hepatopancreatobiliary disease such as pancreatic insufficiency, pancreatitis, cholelithiasis, and CF-related liver disease (CF-LD) (3, 5, 6). Prior studies have shown that there is a high GI symptom burden—particularly bloating, early satiety, and abdominal pain—within this population (4, 7–9). Despite this knowledge, the prevalence of GI disorders in pediatric patients with, versus without, CF is unknown, and GI disorder prevalence has been poorly characterized in children with CF (1, 2).

The prevalence of GI diagnoses and medical therapies and the frequency of diagnostic and therapeutic interventions in this population have yet to be explored on a multisystem scale. Current knowledge is largely focused on GI symptoms rather than diagnoses (7, 8). Therefore, the aim of the present study was to describe, among pediatric PwCF, the prevalence and types of 1) GI disorders, 2) GI medication use, and 3) GI procedural interventions, as compared to a cohort of age-matched children without CF.

## Methods

This study was approved by the Johns Hopkins Medicine Institutional Review Board.

### The TriNetX database

We performed a nested case–control analysis using the global health data platform TriNetX, a deidentified web-based tool for research population cohort and feasibility queries that provides access to EMR-based clinical data from 67 healthcare organizations (HCOs) worldwide. The TriNetX database provides real-time access to longitudinal EMR information, derived from both inpatient and outpatient encounters. TriNetX performs recurring refreshers and spot checks of the data on a monthly basis, with varying data from HCOs.

### Cases and controls

We included all patients between the ages of 0 and 21 years (inclusive) between 1 January 2010 and 1 January 2020. Those with a history of solid-organ transplants were excluded due to the greater complexity of these patients and a greater likelihood of biasing results. Out of this cohort, cases were defined as patients with an ICD-9 or ICD-10 diagnosis code for CF (Supplement). Controls were defined as those patients without a diagnosis of CF.

### Statistical methods

Comparisons between the CF and non-CF cohorts were done using a chi-square test or Fisher’s exact test where appropriate. Bonferroni correction was used due to multiple comparisons, and a *p*-value of <0.0001 was considered significant. Demographic and clinical characteristics were summarized using descriptive statistics. Means with standard deviations or medians with interquartile ranges were used for continuous variables. Counts and percentages were used for categorical variables. GI manifestations in pediatric PwCF were categorized as intestinal, hepatobiliary, or pancreatic disease. Analyses were conducted using the TriNetX platform and SAS version 9.4.

## Results

### Demographics

Query of the TriNetX Research Network dataset identified a total of 81,126 PwCF, including 19,979 pediatric patients ≤21 years of age. Of these, 7,649 met our inclusion criteria for cases, compared to 22,516,240 patients without CF (controls). There was a statistically significant difference in cases versus controls for age, race, and ethnicity as seen in **Table 1**.

**Table 1:** Demographic data.

Variable	Children with CF N (%)	Children without CF N (%)
Total number	7,649	22,516,240
*Mean age in years (SD)	13 (5)	12 (5)
Gender Male Female	4,054 (53%) 3,595 (47%)	11,708,445 (52%) 10,807,795 (48%)
*Race White Unknown Race Black for African American American Indian/ Asian/ Native Hawaiian	2,664 (73%) 1,453 (19%) 535 (7%) 76 (1%)	9,907,146 (44%) 8,781,334 (39%) 2,927,111 (13%) 900,650 (4%)
*Ethnicity Not Hispanic or Latino Hispanic or Latino Unknown ethnicity	5,431 (71%) 1,300 (17%) 918 (12%)	10,357,470 (46%) 9,231,658 (41%) 2,927,111 (13%)
Minimum Age	3	0
Maximum Age	21	21
Median Age	13 (5)	12 (5)

* in the table denotes a p value <0.0001.

### High burden of GI disorders in PwCF

The prevalence of GI disorders was significantly higher among children with CF (**Table 2**). GI disorders were diagnosed in 5,556 of pediatric PwCF (72%), compared to 120,804 controls (0.54%) (*p* < 0.0001). Similarly, the prevalence of each GI disorder subtype—pancreatic, hepatobiliary, and intestinal—was significantly higher among children with CF. Among pancreatic disorders in pediatric PwCF, *exocrine pancreatic insufficiency* was the most common (31%), followed by *chronic pancreatitis* (19%). Among hepatobiliary disorders, *liver disease unspecified* (4.7%) and *other specified disease of the liver* (3%) were the most common. For intestinal and luminal disorders, *constipation* was the most common at 38%, followed by *gastroesophageal reflux disease* (GERD) at 30%.

Table 2Frequency of gastrointestinal manifestations, medications, and procedures within a sample of pediatric patients aged 0–21 diagnosed with and without cystic fibrosis from 2010 to 2020 obtained from the TriNetX database.

**Table:** 

Disease category	Diagnosis [ICD 10 codes]	N=Number of CF patients with diagnosis (Percentage of sample)	N=Number of non-CF patients with diagnosis (percentage)
Disorders of the gastrointestinal tract	Disorders of the gastrointestinal tract [K00-K95]	5,556 (72.6%)	120,804 (0.5%)
	Disorders of gallbladder, biliary tract and pancreas [K80-87]	3374 (43.3%)	2,298 (0.01%)
	Diseases of the liver [K70-K77]	690 (8.9%)	2537 (0.01%)
	Other diseases of the esophagus, stomach, duodenum [K20-31]	2,613 (34.2%)	34,841 (0.2%)
	Other diseases of intestines [K55-K65]	3,307 (43.2%)	49,108 (0.2%)
Pancreatic disease	Other specified disease of the pancreas [K86.8]	3,596 (46.2%)	156 (0.0007%)
	Exocrine Pancreatic insufficiency [K86.81]	2,450 (31.5%)	57 (0.0003%)
	Other Chronic pancreatitis [K86.1]	1,481 (19%)	88 (0.0004%)
	Pancreatic steatorrhea [K90.3]	241 (3.1%)	10 (0.0000%)
	Acute Pancreatitis [K85]	189 (2.4%)	365 (0.0016%)
	Acute pancreatitis unspecified [K85.9]	177 (2.3%)	318 (0.0014%)
	Other Acute pancreatitis [K85.8]	48 (0.1%)	30 (0.0000%)
	Idiopathic pancreatitis [K85.0]	25 (0.3%)	30 (0.0000%)
	Biliary pancreatitis [K85.1]	10 (0.1%)	60 (0.0003%)
Hepato-biliary disease	Liver disease unspecified [K76.9]	363 (4.7%)	335 (0.0015%)
	Other specified disease of the liver [K76.8]	235 (3.0%)	673 (0.0030%)
	Fatty (change of) liver, not elsewhere classified [K76.0]	209 (2.7%)	1216 (0.0054%)
	Fibrosis and Cirrhosis of the liver [K74]	137 (1.8%)	189 (0.0008%)
	Obstruction of the bile duct [K83.1]	68 (0.9%)	192 (0.0008%)
	Other specified diseases of the biliary tract [K83.8]	82 (1.1%)	276 (0.0012%)
	Cholelithiasis [K80]	108 (1.4%)	1,047 (0.0046%)
	Other and unspecified cirrhosis of the liver [K74.6]	84 (1.1%)	105 (0.0005%)
	Hepatic Fibrosis [K74]	77 (1%)	103 (0.0005%)
Luminal diseases	Constipation [K59.0]	2,934 (37.7%)	39,310 (0.2%)
	Gastroesophageal reflux disease [K21]	2,417 (31%)	21,482 (0.1%)
	Gastro-esophageal reflux disease without esophagitis [K21.9]	2,303 (29.6%)	18,905 (0.1%)
	Gastro-esophageal reflux disease with esophagitis [K21.0]	210 (2.7%)	2,133 (0.01%)
	Gastrostomy status [Z93.1]	1,072 (13.8%)	1,819 (0.1%)
	Protein-calorie malnutrition of moderate and mild degree [E44]	716 (9.4%)	961(0.0043%)
	Unspecified protein-calorie malnutrition [E46]	577 (7.5%)	845 (0.0038%)
	Unspecified protein-calorie malnutrition severe [E43]	313 (4.1%)	590 (0.0026%)
	Other specified nonspecific gastroenteritis and colitis [K52.8]	699 (9%)	16,619 (0.1%)
	Allergic and dietetic gastroenteritis and colitis [K52.2]	577 (7.4%)	9,090 (0.4%)
	Intestinal malabsorption, unspecified [K90.9]	544 (7%)	579 (0.0026%)
	Noninfective gastroenteritis and colitis, unspecified [K52.9]	479 (6.2%)	20,116 (0.1%)
	Gastrostomy complications [K94.2]	494 (6.3%)	729 (0.0032%)
	Other and unspecified intestinal obstruction [K56.6]	382 (4.9%)	494 (0.0022%)
	Other malabsorption due to intolerance [K90.4]	291 (3.7%)	1,953 (0.0087%)
	Melena [K92.1]	243 (3.1%)	3,333 (0.01%)
	Other impaction of intestine [K56.4]	189 (2.4%)	777 (0.0035%)
	Esophagitis [K20]	183 (2.4%)	1,518 (0.0067%)
	Other intestinal malabsorption [K90.8]	182 (2.3%)	686 (0.0030%)
	Functional Dyspepsia [K30]	160 (2.1%)	6,489 (0.03%)
	Ileus unspecified [K56.7]	160 (2.1%)	716 (0.0032%)
	Rectal Prolapse [K62.3]	149 (1.9%)	191 (0.0008%)
	Unspecified chronic gastritis [K29.5]	147 (1.9%)	1,312 (0.0058%)
	Gastritis, unspecified [K29.7]	122 (1.6%)	3,037 (0.01%)
	Viral intestinal infections [A08.4]	148 (1.9%)	7,180 (0.03%)

**Table:** 

Medication category	Medication [RxNorm]	N=Number of patients with diagnosis (percentage)	N=Number of patients with diagnosis (percentage)
Digestants	Digestants [GA500]	3,608 (46.3%)	2,299 (0.01%)
	Protease [8031]	3,542 (45.5%)	147 (0.0007%)
	Lipase [6406]	3,513 (45.1%)	1,965 (0.0087%)
	Amylase [743]	3,476 (44.6%)	1,218 (0.0054%)
Laxatives	Laxatives [GA200]	3,569 (45.8%)	43,715 (0.2%)
	Hyperosmotic Laxatives [GA202]	3,441 (44.2%)	38,572 (0.02%)
	Rectal, laxatives [RS300]	1,264 (16.2%)	18,612 (0.08%)
	Stimulant laxatives [GA204]	1,001 (12.9%)	11,325 (0.05%)
	Stool softener [GA205]	528 (6.8%)	8,611 (0.04%)
	Bulk-forming laxatives [GA201]	214 (2.8%)	954 (0.0042%)
	Lubricant laxatives [GA202]	33 (0.42%)	51 (0.0002%)
Gastric medications	Gastric medications [GA900]	3,341 (42.89%)	29,571 (0.13%)
	Omeprazole [7646]	1,701 (21.8%)	12,891 (0.06%)
	Lansoprazole [17128]	1,663 (21.4%)	3,540 (0.02%)
	Esomeprazole [783742]	631 (8.10%)	2,539 (0.01%)
	Antacids [GA100]	1,473 (18.9%)	16,597 (0.07%)
	Sodium bicarbonate containing antacids [GA110]	1,064 (13.7%)	8,160 (0.04%)
	Calcium containing antacids [GA105]	480 (6.2%)	3,387 (0.02%)
	Magnesium containing antacids [GA108]	338 (4.3%)	6,278 (0.03%)
	Aluminum containing antacids [GA101]	176 (2.3%)	76 (0.0003%)
	Histamine Antagonists [GA301]	1,095 (14.1%)	13,963 (0.06%)
	Sucralfate [10156]	193 (2.5%)	1,556 (0.01%)
	Ursodeoxycholate [67427]	758 (9.7%)	439 (0.0019%)
Antidiarrheal	Antidiarrheals	563 (7.2%)	3,442 (0.02%)
	Lactoabacillus acidophilus [6205]	451 (58%)	1,168 (0.0052%)
Mucolytics	Acetylcysteine [197]	269 (3.5%)	5,222 (0.02%)
Vitamins	[RxNorm] [VT000]	3,673 (47.2%)	37,579 (0.2%)
Fat soluble	Vitamin D [VT500]	3,020 (38.8%)	16,992 (0.08%)
Fat soluble	Vitamin K [VT700]	1,779 (22.8%)	6,715 (0.03%)
Water soluble	Vitamin B [VT100]	1,259 (16.2%)	17,585 (0.08%)
Water soluble	Vitamin C [VT400]	785 (10%)	8,322 (0.04%)
Fat soluble	Vitamin E [VT600]	724 (9.3%)	5,069 (0.02%)

**Table:** 

Procedure	Name of service [CPT]	N=Number of patients with diagnosis (percentage)	N=Number of patients with diagnosis (percentage)
Gastroenterology procedures	Esophogastroduodenoscopy procedures [1021431]	736 (9.4%)	5,155 (0.03%)
	Colonoscopy, flexible [10122231]	222 (2.9%)	1,972 (0.0086%)
	Surgical procedures of the digestive tract [1006964]	219 (2.8%)	21,875 (0.0046%)
	ERCP [10214320	27 (0.35%)	129 (0.0006%)
Manometry	Esophagus, gastroesophageal reflux test [1012772]	34 (0.44%)	149 (0.0007%)
	Anorectal manometry [91122]	28 (0.36%)	133 (0.0006%)
	Esophageal manometry [1014295]	10 (0.13%)	60 (0.0003%)
	Gastric motility [91020]	10 (0.13%)	10 (0.0000%)
	Duodenal manometry [90122]	10 (0.13%)	10 (0.0000%)
Surgical procedure of the digestive system	Esophagogastroduodenoscopy Procedures [10214331]	734 (9.4%)	1,273 (0.0056%)
	Change of Gastrostomy tube, percutaneous without imaging or endoscopic guidance [43760]	283 (3.6%)	352 (0.0016%)
	Introduction, Revision and/or Removal procedure on the abdomen, peritoneum, and omentum [1007986]	206 (2.6%)	518 (0.0023%)
	Colonoscopy, flexible [1022231]	223 (2.9%)	2,019 (0.0089%)
	Replacement of gastrostomy tube, percutaneous [1035170]	154 (2%)	235 (0.0010%)
	Surgical laparoscopy [1007375]	132 (1.7%)	50 (0.0002%)
	Surgical procedure on the intestines (except rectum) [1007422]	169 (2.2%)	536 (0.0024%)
	Endoscopic Retrograde Cholangiopancreatography [1021432]	29 (0.37%)	131 (0.0006%)
Otorhinolaryngologic procedures:			
Insertion	Insertion of Gastric Feeding device [ODH6]	406 (5.2%)	795 (0.0035%)
Excision	Excision of duodenum, via artificial or natural opening Endoscopic [ODB9]	238 (3.1%)	1,327 (0.0059%)
	Excision, stomach [ODB6]	225 (2.9%)	1,258 (0.0056%)
	Excision of esophagus [ODB5]	178 (2.3%)	896 (0.0039%)
Drainage	Drainage of Stomach, via artificial or natural opening Endoscopic [OD968ZX]	167 (2.1%)	457 (0.0020%)
	Drainage of esophagus, via artificial or natural opening Endoscopic [OD958ZX]	126 (1.6%)	462 (0.0020%)
	Drainage of duodenum, via artificial or natural opening Endoscopic [OD998ZX]	125 (1.6%)	456 (0.0020%)
Change	Change feeding device in upper intestinal tract, external Approach [OD20]	171 (2.2%)	199 (0.0009%)
Special services	Medical Nutrition Therapy [1013548]	1,768 (22.7%)	5,535 (0.02%)
Consultation	Hospital Inpatient services [1013659	2,202 (28.3%)	23,427 (0.10%)
	Critical Care services [1014309]	402 (5.2%)	4,721 (0.02%)
	Neonatal intensive care services [1019130]	255 (3.3%)	3,084 (0.01%)
Diagnostic radiologic imaging	Ultrasound, abdominal, real-time with image documentation [1010775]	1,327 (17%)	13,606 (0.06%)

The *p*-value for all categories above was <0.0001.

### GI medication use including rectal therapies is higher in PwCF

Digestants were the most common GI medication class used in pediatric PwCF (**Table 2**). Compared to 0.01% of controls, 46% of PwCF were administered digestants. This is consistent with the higher prevalence of constipation seen in pediatric PwCF. *Laxative medications* and *gastric medications* (e.g., proton pump inhibitors, antacids) were the second and third most commonly administered GI medications among pediatric PwCF, consistent with the higher prevalence of constipation and GERD in PwCF.

### GI procedures performed infrequently despite high disease prevalence in PwCF

The prevalence of GI procedures was less than 10% in both groups (**Table 2**). However, PwCF still had significantly higher rates of GI procedures, with the most common being esophagogastroduodenoscopy (EGD) (9.4%) and colonoscopy (2.8%) (*p* < 0.0001).

## Discussion

Pulmonary and pancreatic diseases are typically the hallmarks of CF. In recent years, it has become increasingly clear that PwCF with GI disorders, including but not limited to pancreatic involvement, experience a reduced quality of life (3, 4, 7, 10–12). This has become of greater interest to the CF community as treatment outcomes for lung diseases have been transformed by the advent of highly effective modulator therapy (HEMT) (13). However, multicenter data are sparse on the prevalence and types of GI disorders in PwCF, especially beyond pancreatic disease. In this nested case–control analysis, we found a significantly higher prevalence of GI disorders—and accordingly, GI medication use—among pediatric PwCF when compared to those without CF, further exemplifying why research on CF GI disease is timely and can be transformative in this population. In addition, we determined that while the burden of GI disease is much greater in PwCF, and while GI procedural frequency is also significantly greater in this population, the total proportion of those undergoing procedures remains relatively low (<10%).

### Prevalence of the disease and medication use

Our study highlights the broad and diverse diagnostic codes practitioners use to describe chronic pancreatic and liver diseases, none of which are specific for CF. Our findings of a high prevalence of chronic pancreatic disease (at 53.6%, including exocrine pancreatic insufficiency, chronic pancreatitis, and pancreatic steatorrhea, while 46.2% of patients had received a diagnosis of other specified disease of the pancreas) in pediatric PwCF are concordant with prior knowledge regarding pancreatic disease, indicating a prevalence of up to 85% and its underlying mechanisms in CF (1, 2, 14–18). This database further provides greater specificity to the different liver manifestations in CF while prior studies have previously assumed a broad prevalence of <5%–68% (19), and it also highlights the evolving landscape of CFLD as most recent clinical guidelines recommend stratifying disease as advanced CFLD (aCFLD) versus CF hepatobiliary involvement (CFHBI) (20). The variety of codes used especially in liver disease is reflective of the heterogeneity of this disease and the likely need for a CF-specific ICD-10 code which will be instrumental in future clinical trials (15, 16).

Our findings further demonstrate a high prevalence of constipation among pediatric PwCF and as such more frequent use of digestants and laxative medications. While most laxatives were oral, a surprisingly high proportion of patients needed rectal therapies to help alleviate constipation due to likely a higher severity of disease and due to the risk of distal intestinal obstruction syndrome (DIOS) in CF. Whether or not rectal therapies were more frequently used in the setting of DIOS or routinely for constipation management could not be appreciated through this study and could be a future area of investigation.

Despite GERD being the second most common intestinal diagnosis, correlating with the high use of gastric medications, there was a lower prevalence of esophageal manometry and impedance testing. There is a lack of diagnostic guidelines within the pediatric CF population for GERD, but typically, pH impedance, esophageal manometry, or endoscopy is not indicated unless the patient is resistant to therapy or has alarming symptoms (21). The use of HEMT has improved lung disease, its impact on GERD has not yet been examine. Defining the true prevalence of GERD in this population along with the prevalence of a healthy control group prior to the broad adoption of HEMT use is critical as it allows us to determine in the future if HEMT therapy allows PwCF to have a similar prevalence of GI manifestations with adequate and early therapy (13). Therefore, considering the role HEMT has on improving lung disease, we may better delineate the prevalence of a GERD diagnosis for either 1) true reflux or 2) to aid in the management of malnutrition in PwCF by administering acid blockade for presumed improved enzyme absorption.

The overall prevalence of GI procedures, although thought to be low, was comparably significantly greater in the PwCF compared to the non-CF control sample even with the Bonferroni adjustment due to such a large control sample. Despite the greater risk of sedation in PwCF, procedures were more likely to be performed on these patients again likely due to the significantly higher burden of disease. Endoscopies being more common in patients with CF over controls but still proportionally less common than controls may be secondary to multiple factors including: 1) historically, gastroenterologists were scarcely involved in the care of patients with CF as this was primarily managed by pulmonologists and pediatricians; 2) some of the GI CF manifestations especially reflux and dysmotility are widely accepted in the CF community as part of the disease; and 3) secondary to underlying lung disease and subsequently greater risk of sedation. Thus, it is likely that despite a high burden of disease, the use of procedures is less commonly felt to be needed for diagnosis.

Prior studies have shown a high GI symptom burden for these patients; thus, defining the prevalence of GI-related manifestations is important to develop measures that improve quality of life and define changes in disease state after using highly effective modulators.

## Dataset limitations

We elected to use the TriNetX database despite its limitations for the following reasons. In other known registries such as the CF Foundation Registry, patients must opt-in to be recorded within the database, whereas TriNetX includes all recorded patients in a healthcare system. Furthermore, the TriNetX database collects data not yet available in the CF Foundation Registry and allows for comparison against an age-matched cohort. For our description, we relied on the search for GI diagnoses by diagnostic codes. Thus, patients may be diagnosed inaccurately, non-specifically, or by physicians of differing specialties, introducing further variability. To prevent this, diagnoses were reviewed by a pediatric CF gastroenterologist during analysis. Additionally, the TriNetX system includes an ICD-9 to ICD-10 mapping based on the General Equivalence Mappings (GEMs). GEMs use custom algorithms and curation to transform data from ICD-9-CM to ICD-10-CM. This allows diagnostic terms from historic data and HCOs who provide ICD-9-CM terms to be treated according to query. The time period—1 January 2010 to 1 January 2020—was chosen to include a full decade of data while balancing the exclusion of potential influence from COVID-19 and HEMT use. This may contribute to the findings of exocrine pancreatic insufficiency having a prevalence of 31% in the CF population when it is known to exist in greater than 85% of pediatric CF (22). Furthermore, while we anticipated detecting diagnoses such as small bacterial overgrowth in the dataset, this was not reported. There is also no dedicated ICD-10 code for cystic fibrosis-associated liver disease, which is a heterogeneous disease. Thus, the involvement of the liver in CF may be unknown with subclinical features until the patient presents with diffuse non-specific liver damage. Comparing patients with versus without CF was chosen to reduce complexity; however, other chronic diseases may contribute to the results. Lastly, this is a descriptive study and, thus, does not demonstrate causation of GI manifestations in CF.

Notwithstanding these limitations, our findings demonstrate that the prevalence of GI disorders, medication use, and procedure performance is greater in pediatric PwCF compared to those without CF.

## Conclusion

This study uniquely describes the strikingly high prevalence of GI manifestations and associated GI medication use among pediatric PwCF internationally as compared to a large healthy control. Interestingly, we saw a comparatively lower prevalence of GI procedures in this population despite the high burden of disease, though still substantially greater when compared to controls. These results highlight the broad array of GI manifestations associated with CF, suggesting a need for further focus on these disorders in the new era of HEMT. This study better defines the complexities of GI disease including the prevalence of GI disease, medication use, and interventions prior to broad and disseminated use of HEMT, which will be critical in understanding the effects of highly effective modulator therapy on the full scope of CF manifestations.

## Data Availability

The original contributions presented in the study are included in the article/supplementary material. Further inquiries can be directed to the corresponding author.
